# Case reports with literature review of an aneurysmal bone cyst in the maxilla and mandible of two juvenile dogs

**DOI:** 10.3389/fvets.2025.1632403

**Published:** 2025-07-31

**Authors:** Colin Adley, Mary Krakowski Volker

**Affiliations:** Animal Dental Center, Towson, MD, United States

**Keywords:** aneurysmal bone cyst, maxilla, mandible, dog, canine

## Abstract

This report identifies two cases of juvenile dogs with an aneurysmal bone cyst (ABC). The first case describes an ABC in the left rostral mandible, and the second case describes an ABC in the right maxilla. ABCs are typically identified in juvenile or young animals and have been reported in a variety of species. An ABC results from the intraosseous expansion of soft or immature bone and is more appropriately identified as a pseudocyst due to the absence of an epithelial lining. An ABC is most likely believed to be caused by trauma, which contributes to the formation of an expansile, blood-filled lesion encased with immature, proliferative bone. The classic presentation is an osteolytic, cavitated lesion in the metaphysis region of long bones. Both diagnostic imaging and histopathology are required for the diagnosis of an ABC, with differential diagnoses being fibrosarcoma, osteolytic osteosarcoma, osteoblastic or giant cell tumor, the unilocular membrane-lined simple bone cyst, ameloblastoma, fibro-odontoma, and papillary squamous cell carcinoma. In human medicine, ABCs are found in the head and neck region in 2–12% of reported cases, with 60–70% of the cases occurring in the jaws. Aneurysmal bone cysts in the maxillofacial region of the dog are a rare finding in veterinary medicine and are currently without confirmed etiopathogenesis. The following case reports describe an ABC in the left rostral mandible of a 7-month-old dog treated with complete excision and an ABC in the right maxilla of a 12-week-old dog treated with incisional biopsy and curettage that showed resolution of right facial swelling before being lost to follow-up. These cases and literature review add to the paucity of veterinary literature regarding aneurysmal bone cysts in dogs and provide case descriptions and treatment recommendations for this rare juvenile lesion.

## Introduction

An aneurysmal bone cyst (ABC) is a blood-filled cystic or multicystic osteolytic expansile lesion that is more accurately identified as a pseudocyst due to the absence of an epithelial lining and results from intraosseous expansion of soft or immature bone (1). The space is filled with blood, fibroblasts, and multinucleated giant cells, which form a membrane that migrates over surfaces to create a lining of interior septa that may or may not be seen radiographically (2, 3). An ABC is suspected to be caused by trauma, which contributes to the formation of an expansile, fluid-filled lesion that is encased within immature, proliferative bone (1). Aneurysmal bone cysts are rare lesions identified in dogs and cats. ABCs are typically diagnosed in juvenile or young animals and have been reported in a variety of species, such as humans, llamas, psittacines, cattle, horses, dogs, and cats (4). The classic presentation is an osteolytic lesion in the metaphysis region of long bones. Recently, a case was reported of an ABC located in the right maxilla of a 4-month-old male dog surrounding an unerupted right maxillary fourth premolar tooth and an unerupted right maxillary first molar tooth (5). In human medicine, the first use of the term aneurysmal bone cyst was reported in 1942, and the literature has shown a 2–12% prevalence in bones of the maxillofacial region (6). The two cases reported here will present the rare diagnosis of an ABC diagnosed in the mandible of a 7-month-old dog and in the maxilla of a 12-week-old dog. This report and literature review are intended to broaden the differential diagnoses of osteolytic lesions in the maxillofacial region of juvenile dogs, with an understanding of treatment options when these cases are presented to veterinarians.

## Case reports

### Case 1

**Figure 1:**
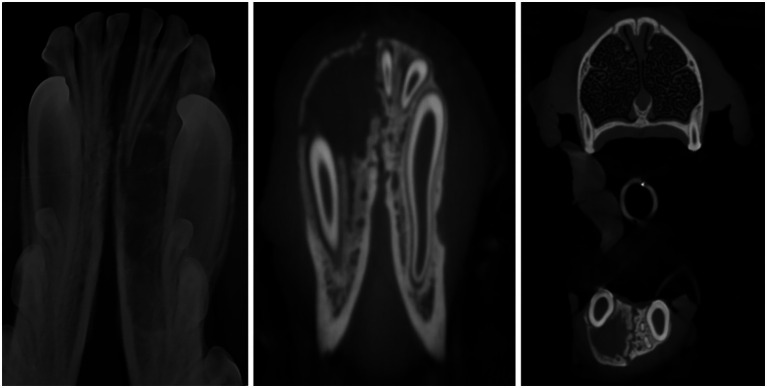
Radiograph and CT images showing aneurysmal bone cyst in a 7-month-old Irish Wolfhound. Osteolysis extending and encompassing the left rostral mandible from the mandibular symphysis with the left mandibular canine tooth displaced buccally. The left mandibular incisors are missing from the CT as they were extracted at time of incisional biopsy.

A 7-month-old, 54-kg, female intact Irish Wolfhound was examined by a private veterinary dentistry and oral surgery referral practice for evaluation of a class II malocclusion with linguoverted mandibular canine teeth in February 2020. The mandibular canine teeth were traumatically occluding at the palatal aspect of the maxillary canine teeth, resulting in moderate indentation/traumatic palatal ulceration. Crown reduction and vital pulp therapy of the mandibular canine teeth were recommended as the best treatment option for the dog’s traumatic malocclusion, and the procedure was scheduled for 2 weeks later. The dog’s procedure was then performed 2 weeks later, and on anesthetized oral examination with full-mouth dental radiographs, an incidental, large rostral mandibular osteolytic lesion was diagnosed. The osteolysis extended from the apex of the left mandibular canine tooth rostrally and included the left mandibular incisor teeth (Figure 1). Incisional biopsies were taken by surgically extracting the left mandibular incisor teeth as they were mobile; this enabled surgical removal and sampling of the tissue lining associated with the osteolytic bony lesion using a curved periosteal elevator via a curettage technique. These tissue biopsies were submitted to the Antech Laboratory Oral Pathology Team for histopathology. The surgical biopsies were reported as islands of amorphous basophilic material (negative for amyloid) surrounded by fibroblasts, epithelioid macrophages, multinucleated giant cells, acute hemorrhage, and fibrosis surrounding mineralized material, which merges with anastomosing trabeculae of woven bone lined by a single layer of prominent osteoblasts, consistent with an aneurysmal bone cyst. Treatment options of surgical curettage, en bloc resection (marginal excisional biopsy), and bilateral rostral mandibulectomy (wide excisional biopsy) were discussed with the dog’s owner. Due to the degree of osteolysis, displacement of dentition, a literature report of malignant transformation of an aneurysmal bone cyst to a chondrosarcoma in a large breed dog, and uncertainty of the accuracy of the incisional biopsy report due to the rarity of ABC diagnosis, bilateral rostral mandibulectomy with wide excisional margins was elected by the dog’s owner to prevent any chance of recurrence and minimize financial concerns (7). The dog was then scheduled for a conventional computed tomography (CT) scan with contrast and surgical removal of the aneurysmal bone cyst, with submission to histopathology to confirm diagnosis 4 weeks later. The CT scan findings, reported by a board-certified veterinary radiologist, were an expansile lytic lesion of the rostral left mandible that extended rostromedially from the level of the mandibular symphysis and caudally/distally to the level of the root of the left mandibular canine tooth. There was moderate thickening of the surrounding cortical margin. The radiology report conclusion was expansile lytic mass of the rostral left mandible consistent with an aneurysmal bone cyst. The lesion extended across the mandibular symphysis, causing pressure necrosis to the medial aspect of the right rostral mandible without complete invasion (Figure 1). At the time of the initial biopsy, differential diagnoses based on review of current literature included chondrosarcoma, fibrosarcoma, osteogenic sarcoma, hemangiosarcoma, osteolytic osteosarcoma, osteoblastic or giant cell tumor, simple bone cyst, and aneurysmal bone cyst (4, 7, 8). A bilateral rostral mandibulectomy was performed from the left mandibular second premolar through the right mandibular canine tooth with the intent of resection with 10 mm margins. The resection was submitted for histopathology, and the diagnosis was confirmed as a completely excised aneurysmal bone cyst. Final histopathology results were submitted and reviewed by two separate pathology departments and confirmed as a completely excised aneurysmal bone cyst. The dog recovered well from the procedure. The owner was contacted 1 year postoperatively and declined a 1-year follow-up appointment for repeat evaluation with diagnostic imaging. The owner did report the dog was doing well without overt visible recurrence of the ABC.

### Case 2

**Figure 2:**
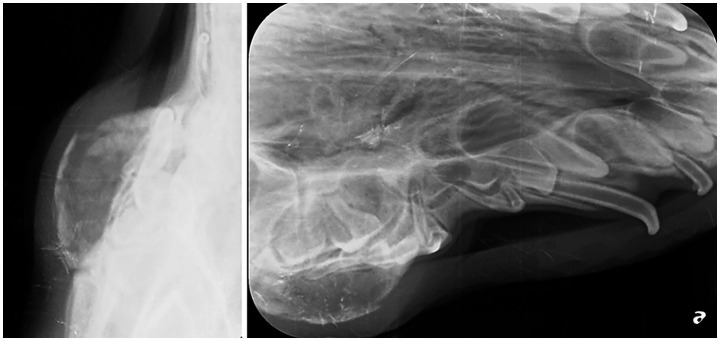
Radiographic image of a right maxillary aneurysmal bone cyst. Expansive thin lining of the bone with osteolysis at the level of the deciduous right maxillary third premolar to the deciduous right maxillary fourth premolar.

**Figure 3:**
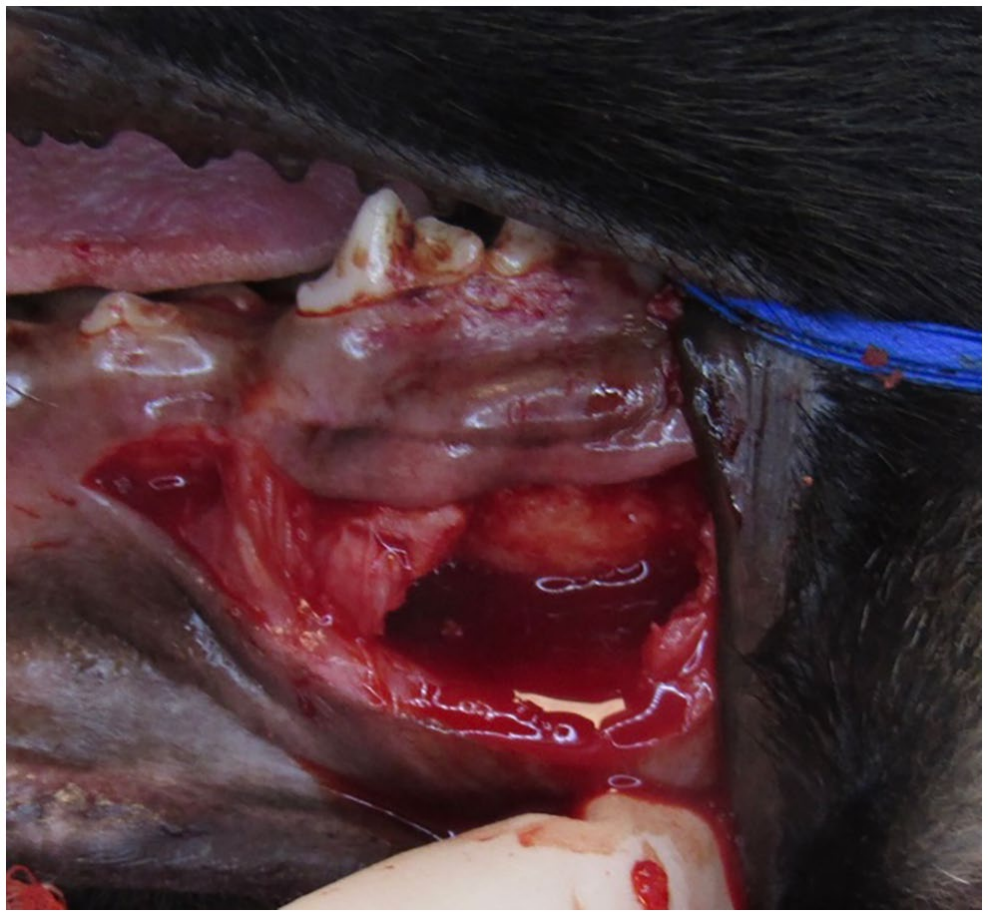
Intraoperative image showing the hemorrhage filled void of an aneurysmal bone cyst in the right maxilla in a 12-week-old German Shepherd Dog.

A 12-week-old, 9.7-kg, female intact, German Shepherd Dog presented for oral evaluation due to an acute onset of right-sided facial swelling in August 2020. The facial swelling had been present for 1 week and was rapidly growing during that period. Oral examination findings included a deciduous dentition with a complicated crown fracture of the deciduous left maxillary third incisor tooth and hypodontia characterized by the absence of the deciduous left maxillary first incisor tooth. Marked, firm swelling of the right maxilla at the location of the deciduous right maxillary third premolar tooth to the deciduous right maxillary fourth premolar tooth was palpated and visualized. The dog was anesthetized, and full-mouth dental radiographs were obtained. At the time of the dog’s procedure, this practice location did not have advanced imaging available on site; thus, only dental radiographs were obtained. The dental radiographs showed hypodontia of the deciduous left maxillary first incisor with unerupted permanent dentition (tooth buds present), and a thin lining of the bone with circular, poorly defined osteolysis at the level of the deciduous right maxillary third premolar tooth to the deciduous right maxillary fourth premolar tooth (Figure 2). A firm, blood-filled, bone swelling was present, and a right maxillary incisional biopsy was obtained through an incision made 3 mm apically to the right mucogingival junction at the deciduous right maxillary second premolar and deciduous right maxillary third premolar tooth (Figure 3). The firm bone swelling was opened with a #1 round carbide bur on a high-speed handpiece, and curettage was performed with a curved #4 periosteal elevator to obtain multiple incisional biopsies and remove the soft tissue lining to be submitted to Specialty Oral Pathology for Animals for histopathologic review. Histopathology diagnosed the lesion as immature bone with localized loss of architecture, hemorrhage, proliferative mesenchymal membranes, and giant cells consistent with aneurysmal bone cyst. The dog returned for a follow-up 2-week postoperative physical exam, and the right maxillary swelling had completely resolved. An anesthetic recheck with cone beam CT scan was offered, but the owner declined due to the dog’s clinical improvement, and the dog was unfortunately lost to the follow-up procedure when contacted 6 and 12 months postoperatively.

## Discussion

Because ABCs are rare in the maxillofacial region of dogs, it is important to understand how ABCs are diagnosed and treated in human medicine. Human literature first described the aneurysmal bone cyst as “homerus ossifying haematoma” in 1893, but “aneurysmal bone cyst” was adopted in 1942 (9). Since then, ABCs have been identified in almost every part of the human skeleton, with more than 50% occurring in long bones and in the vertebral column. The first descriptions of maxillofacial ABCs were reported as two mandibular cases in 1958 (10). A multicenter retrospective cohort study of patient charts from 1967 to 2013 showed that ABCs were significantly more common in the mandible than in the maxilla, with the posterior region of the maxilla and mandible being more common than in the anterior region. ABCs generally tend to affect adolescents younger than 20 years old (11) with a slight predilection for females over males (12). ABCs are present anywhere from 2 to 12% in the head and neck region, with 60–70% of the cases being reported in the jaws. ABCs represent 1.5% of non-odontogenic and non-epithelial cysts of the jaw (13). ABCs occur with an estimated incidence in the human population of approximately 0.14–0.32 per 100,000 individuals. Similar to reported cases in veterinary literature, ABCs most commonly occur in the metaphyseal region of long bones in people (14).

Multiple hypotheses have been made to determine the etiopathogenesis of ABCs without being widely accepted. Theories range from a post-traumatic insult, reactive vascular malformation, and genetically predisposed bone tumors (15). Regarding trauma, some hypotheses have suggested that trauma results in subperiosteal regions or incompletely repaired intramedullary hemorrhage (16). Another suggested etiopathogenesis is an increase in venous pressure and vascular engorgement originating from hemodynamic disturbances leading to bone resorption and erosion (16). The recent identification of recurrent chromosomal translocations involving the USP6 gene showed that ABCs have clonal neoplastic processes and are present in approximately 65–70% of cases. USP6 is a ubiquitin-specific protease with gene localization to chromosome 17 and has an important role in cell signaling, angiogenesis, and inflammatory response (3). ABCs are described as benign lesions as the USP6 gene has been described in other benign entities, including nodular fasciitis, myositis ossificans, fibro-osseous pseudotumor of digits, and fibroma of the tendon sheath, all sharing histopathological findings with ABC (3). ABCs used to be classified as primary ABC or secondary ABC, but the new nomenclature now classifies the lesions as “ABC” and “ABC-like changes.” ABCs are considered a primary bone lesion in 70% of cases, whereas the remaining 30% of cases represent ABC-like changes associated with different primary bone tumors. The most common tumors associated with ABC-like changes were giant cell tumor and chondroblastoma (3). Neither of the two canine cases reported here was known to have experienced trauma prior to presentation, and the cause of the ABC in both cases is unknown.

ABCs in human radiography appear as an expansive, osteolytic, unilocular or multilocular radiolucent lesion, with expansion and thinning of the surrounding cortical bone. This variegated appearance in imaging could be due to the three separate types of ABCs (17) described below. Internal septations may or may not be visible, and ABCs have different types of periosteal reactions (13). In an assessment of 120 maxillofacial ABCs, all cases showed radiolucent unilocular or multilocular patterns (12). Another analysis of 17 ABCs of the jaws showed that teeth involved with the ABC will still be vital; however, the teeth may be displaced and eventually reveal concomitant external root resorption (15). In human diagnostic imaging, the use of CT and MRI scans is commonly used in conjunction to help identify ABCs. CT has the advantage of identifying areas of mineralization, cortical destruction, and periosteal reaction, while MRI scans have a better contrast resolution to allow a precise delineation, identifying perilesional edema, internal composition, and different signal intensity of fluid-filled levels (3). Despite advances in diagnostic imaging, only histopathological findings can provide 100% certainty in an ABC diagnosis. Radiology, MRI, and CT may be suggestive but are not sufficient alone to confirm an ABC diagnosis without histopathology (6).

The World Health Organization defines an ABC as a benign intraosseous lesion, characterized by blood-filled spaces of varying size associated with a fibroblastic stoma containing multinucleated giant cells, osteoid, and woven bone that can only be diagnosed by histopathological features of the whole lesion (18). ABCs are considered pseudocysts due to the absence of an epithelial wall and are separated into 3 types based on histopathological features as well as clinical presentations (18). The three types are: conventional ABC (or vascular variant), making up 95% of cases; solid ABC, making up 5% of cases; and mixed (15). The conventional ABC features are described extensively above. The solid ABC is described as an unusual solid, non-cystic, focally hemorrhagic, firm to soft, gray-white bone lesion with fibroblastic, osteoblastic, osteoclastic, aneurysmal, and fibromyxoid elements, and mixed ABCs lie between the two previous variants (13, 18). To the authors’ knowledge, only the conventional ABC has been reported in dogs.

Human differential diagnoses for ABCs are other multilocular lesions such as ameloblastoma, epithelial cysts, ossifying fibroma, giant-cell granulomas, or sarcomas (9). A malignant telangiectatic osteosarcoma should be included in the differential diagnosis in cases of aggressive and destructive behavior with soft tissue invasion. While telangiectatic osteosarcomas only account for <5% of human osteosarcomas, these tumors have similar radiographic and histological features (10). For this reason, confirming a diagnosis of an ABC is extremely important in determining proper treatment. In animals, the differential diagnoses include fibrosarcoma, osteolytic osteosarcoma, osteoblastic or giant cell tumor, ameloblastoma, and the unilocular membrane-lined simple bone cyst (8). Of note, there is a reported case of an ABC undergoing malignant transformation to a chondrosarcoma in the ulna of a dog requiring forelimb amputation after an initial en bloc (marginal) resection with cancellous bone graft (7). The authors thus recommend periodic imaging monitoring of ABC cases treated with conservative curettage or en bloc resection to confirm complete resolution.

Surgery is the treatment of choice for ABCs in the jaws in human medicine. Conservative surgical methods, such as curettage, are preferred for young patients, considering that ABCs are benign lesions. However, as ABCs are often multilocular, curettage alone is difficult, and multiple treatment procedures including curettage, excision, cryotherapy, block resection, bone grafting, and open packing are often utilized (13, 18). Simple curettage has a recurrence rate of 21–50% while radical resection methods show a recurrence rate of 11–25%; recurrence most frequently occurs within the first year post-initial treatment (18). Treatment should depend on the size and location of the cyst. Aggressive lesions with painful symptoms, progression, and osteolysis should be resected completely (9). A case report of an ABC of the mandibular condyle showed that a complete resection of the coronoid and condylar processes that was reconstructed with a costochondral rib graft and plate provided no recurrence at 5 years with normal occlusion (19).

While ABCs are an uncommon incidental finding in juvenile dogs, it is important for ABCs to be a differential diagnosis for osteolytic maxillofacial lesions in juvenile dogs. The two cases presented here have analogous diagnostic imaging and histopathology findings, albeit their anatomic locations are different. While the metaphyseal region of long bones is the most common location of an ABC, ABCs have also been reported in the vertebrae, pelvis, ribs, and scapula of dogs (4, 7, 20). Computed tomography of ABCs located within the metaphysis of long bones in a dog has shown soft tissue-blood density within an expansile lytic bone lesion (8). Radiographic appearance of ABCs is typically expansive, eccentric, and osteolytic (8). The expansile lytic bone lesion can have focal areas of lucency resembling ‘soap bubbles’ divided by bony septa and surrounded by a thin shell of periosteal new bone (7). These imaging findings can be seen in the diagnostic images of both cases 1 and 2 (Figures 1, 2). Case 2 shows a thin lining of the bone with circular, poorly defined osteolysis at the level of the deciduous right maxillary third premolar to the deciduous right maxillary fourth premolar (Figure 3).

ABCs have been diagnosed in multiple other animal species (4). The radiographic findings of ABCs in cats are similar to those in dogs. An ABC reported in the scapula of a cat showed a bone obliterated with marked septated multilocular expansile lysis without evidence of normal bone but leaving the humoral head unaffected (4). Ultrasonographic evaluation of the lesion revealed multiple cavitary zones filled with moderately echogenic fluid containing slow-moving particles that were thought to be consistent with blood (4). MRI imaging showed a multiloculated mass with a central fluid-filled cavity irregularly and thinly marginated with multiple, smaller fluid-filled cavities containing sediment (4). Radiographic imaging of an ABC in the metatarsal bone of a cat showed lytic enlargement of the bone with cortical thinning, and CT imaging revealed an expansile osteolytic lesion with a periosteal reaction (20). In the two canine cases reported here, both were imaged with full mouth dental radiographs, and case 1 received a conventional CT. At the time of surgery for both patients, Cone Beam CT (CBCT) was not available at the practice, and case 2 was lost to follow-up when CBCT imaging became available. Advanced imaging (conventional CT with contrast and/or CBCT) of an ABC is recommended by the authors to further characterize the appearance and extent of the osteolytic lesion and for treatment planning purposes. Case 1 treatment planning was based on the dental radiograph and conventional CT imaging of the dog; conventional CT allowed for further understanding of the extent of the lesion and margin resection. In general, an ABC should be included in the differential diagnosis of a juvenile patient with an osteolytic, expansive, unilocular or multilocular mass.

**Figure 4:**
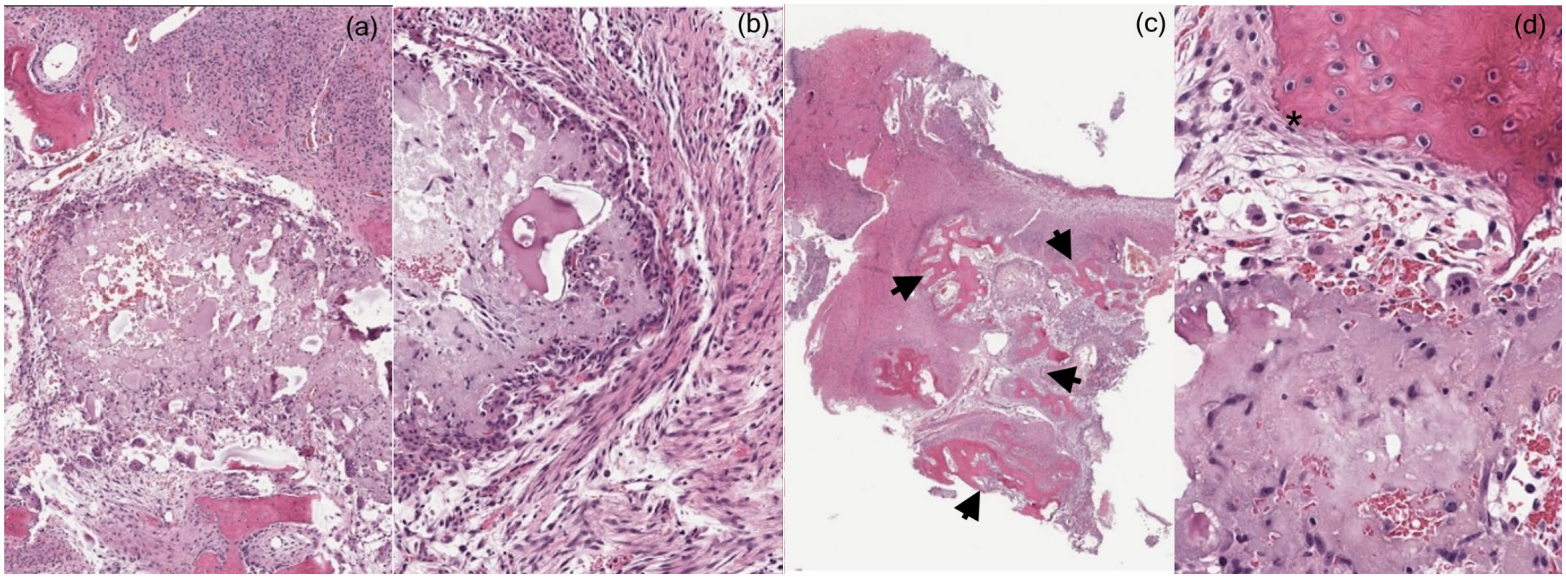
**(a,b)** Histopathologic findings show multiple, variably sized, islands of amorphous basophilic material surrounded by fibroblasts, epithelioid macrophages and multinucleated giant cells. **(c)** Admixed with the mineralized material, there is a variable amount of acute hemorrhage. Moderate amount of fibrosis surrounding the mineralized material merging with anastomosing trabeculae of woven bone (arrows). **(d)** The trabeculae of woven bone are lined by a single layer of prominent osteoblasts (asterisk). Marked woven bone proliferation and mineralized material with multinucleated giant cells is consistent with an aneurysmal bone cyst.

The histological features of ABCs have been consistent between dogs and cats, independent of the anatomical location. An ABC in the ulna of a dog was described as periosteal tissue in continuity with bone trabeculated tissue; trabecular bone with notched margins; osteoblasts and osteoclasts consistent with osteolysis; bone marrow cavity with fibroblasts and connective tissue; trabecular margins covered by osteoblast line, with bone matrix deposition, and hemorrhagic cavities delineated by fibroblasts and rare osteoblasts (21). Fluid-filled cavities have been shown to have many blood cells, osteoclast-like multinucleated giant cells, fibroblasts (4), macrophages (8), osteoid lined by angular polygonal cells, and mature collagenous stroma (4). The Irish wolfhound in case 1 had the histological description of marked woven bone proliferation and mineralized material with multinucleated giant cells (Figure 4). The German Shepherd Dog in case 2 had a histological description of immature bone with localized loss of architecture, hemorrhage, proliferative mesenchymal membranes, and giant cells.

Due to ABCs typically being localized in the metaphysis of long bones, limb-sparing treatment is favored as the first option. Curettage with bone grafting is the recommended initial surgical treatment of an ABC in the metaphysis of long bones. More advanced surgical treatment, including complete excision or amputation, is usually recommended if the cyst is causing lameness, swelling, or pain because of compression of the surrounding soft tissues (20). The prognosis is excellent in human patients and animals when complete excision is feasible (4, 7, 8, 20). Because complete excision of ABC was reported to have an excellent prognosis, and due to financial constraints and unwillingness to have additional treatment performed in the future in case of recurrence, the owner of the Irish Wolfhound in case 1 elected bilateral rostral mandibulectomy as the dog’s treatment. The dog was reported alive and doing well 1 year postoperatively without the recurrence of the ABC. Because the dog in case 2 was lost at follow-up, the initial biopsy with surgical curettage of the right maxilla was suspected to be successful; however, long-term follow-up was not available. Based on this case report and literature review, the authors recommend initial full-mouth dental radiographs, CT, and/or CBCT if available, with incisional biopsy curettage to remove the entire soft tissue lining of a suspected ABC for histopathology review. A recheck to examine the incisional biopsy healing should occur 2 weeks after the initial procedure. If an ABC is diagnosed, imaging is recommended 4–6 months after the initial procedure (dental radiographs and CT and/or CBCT). If the ABC has not resolved, characterized by bone healing and filling of the defect, on follow-up imaging, then more aggressive surgical treatment characterized by marginal to wide excisional biopsy is recommended.

### Summary

Aneurysmal bone cysts (ABCs) are a benign, osteolytic lesion found in the maxillofacial region of the dog and are a rare finding in veterinary medicine. There is no confirmed etiopathogenesis for ABCs. In human medicine, there are multiple etiopathogenesis hypotheses, with a history of trauma being slightly favored, yet these hypotheses remain unproven. In veterinary medicine, a diagnosis of an ABC is dependent on histopathology, and advanced imaging can aid in the development of differential diagnoses and treatment planning. The treatment of an ABC is surgical and highly dependent on the size and location of the lesion. If complete curettage is not possible after the diagnosis of an ABC, the treatment of choice would be complete resection of the ABC. While uncommon, ABCs should be a differential diagnosis in juvenile patients when a multilocular, expansive bone lesion is noted on diagnostic imaging.

## Data Availability

The original contributions presented in the study are included in the article/supplementary material, further inquiries can be directed to the corresponding author.
